# Characterization of the complete mitochondrial genome of the Seron yak (*Bos grunniens*)

**DOI:** 10.1080/23802359.2019.1627941

**Published:** 2019-07-11

**Authors:** Xian Guo, Xiaoyun Wu, Pengjia Bao, Min Chu, Xuezhi Ding, Lin Xiong, Chunnnian Liang, Jie Pei, Ping Yan

**Affiliations:** Key Laboratory of Yak Breeding Engineering of Gansu Province, Lanzhou Institute of Husbandry and Pharmaceutical Sciences, Chinese Academy of Agricultural Sciences, Lanzhou, People’s Republic of China

**Keywords:** Domestic yak, maximum-likelihood, Illumina sequencing, iterative mapping, mitogenome

## Abstract

In this study, we assembled the mitochondrial genome for Seron yak (*Bos grunniens*), a local yak breed with strong adaptation to marshy grasslands. The resultant mitochondrial genome is 16,325 bp long with an A + T-biased base composition (61.0% A + T) and harbours the typical set of 37 mitochondrial genes and 1 non-coding control region. The PCGs start with the typical ATA or ATG codons and are terminated with TAA, TAG or the incomplete stop codon T. Phylogenetic analysis supports the inclusion of *Bison* within the genus *Bos*, and suggests that Seron yak is most closely related to Datong yak and polled yak.

Seron yak (*Bos grunniens*) is a breed of domestic yak with its distribution mostly confined to Henan County, Huangnan Tibetan Autonomous Prefecture, Qinghai Province, China, and is well-known for its strong adaptation to marshy grasslands. To date, little is known about its genetics and genomics as well as its relationship with other yak breeds. Here, the complete mitochondrial genome of Seron yak was characterized by high-throughput Illumina sequencing technology. Besides, we also investigated its relationship with its congeners and the closely related genus *Bison* (Guo et al. 2016).

A blood sample of Seron yak was collected from Henan County, Huangnan Tibetan Autonomous Prefecture, Qinghai Province (34°49′ N, 102°14′ E). A voucher specimen is held in the Key Laboratory of Yak Breeding Engineering of Gansu Province, Lanzhou Institute of Husbandry and Pharmaceutical Sciences (Lanzhou, Gansu Province, China). Isolation of genomic DNA was carried out with the QIAamp DNA Blood Mini Kit (Qiagen, CA, USA). Library preparation and high-throughput sequencing were conducted by Annoroad Gene Technology (Beijing, China) following the manufacturer’s protocol of the Illumina HiSeq XTM Ten Sequencing System (Illumina, CA, USA). A total of 24.6 M raw reads of 150 bp were retrieved and used to assemble the mitochondrial genome with MITObim v1.9 (Hahn et al. 2013); the reference sequence (GenBank accession: JQ692071) was previously published by Qiu et al. (2012). Mitogenome annotation was conducted by comparing with those of its congeners.

The mitochondrial genome of Seron yak (GenBank accession: MK780192) is highly similar to those of other yak breeds (Qiu et al. 2012; Chu et al. 2016; Guo et al. 2016; Wu, Chu, et al. 2016; Wu, Ding, et al. 2016). It is 16,325 bp long with an A + T-biased base composition (33.7% A, 25.8% C, 13.2% G & 27.3% T; ‘light strand’), and harbours the typical set of 37 animal mitochondrial genes and 1 control region. The typical start codon ATA was annotated for three protein-coding genes (PCGs) (*ND2*, *ND3*, and *ND5*), and ATG for the remaining ten PCGs (*ATP6*, *ATP8*, *COX1*, *COX2*, *COX3*, *CYTB*, *ND1*, *ND4*, *ND4L*, and *ND6*) codons. Three types of stop codons were annotated, i.e. TAA (*ATP6*, *ATP8*, *COX1*, *COX2*, *CYTB*, *ND1*, *ND4L*, *ND5*, and *ND6*), TAG (*ND2*), and the incomplete stop codon T (*COX3*, *ND3*, and *ND4*). The tRNAs vary in size between 60 (*tRNA-Ser^AGN^*) and 75 bp (*tRNA-Leu^UUR^*) with a total length of 1509 bp. The two rRNAs are 957 bp (*12S rRNA*) and 1571 bp (*16S rRNA*) long, and are separated by *tRNA-Val*. The control region is 895 bp long, and resides between *tRNA-Pro* and *tRNA-Phe*.

Phylogenetic analysis was conducted based on the maximum-likelihood analysis of the concatenated sequences of all 13 mitochondrial PCGs for a group of 17 *Bos* and *Bison* taxa with MEGA7 (Kumar et al. 2016) (Figure 1). Seron yak was found to be most closely related to Datong yak and polled yak. Furthermore, our result appears to support the previously proposed inclusion of *Bison* within the genus *Bos* (reviewed by Douglas et al. 2011).

**Figure 1.**
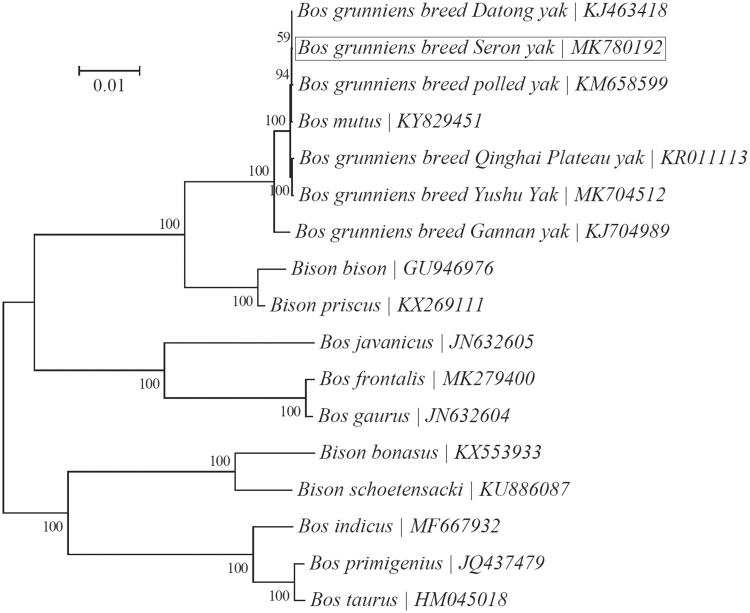
Phylogeny of two related genera *Bison* and *Bos* based on the maximum-likelihood analysis of the concatenated sequences of 13 mitochondrial protein-coding genes (alignment size: 11,370 bp). The best-fit nucleotide substitution model is ‘HKY + G’. The support values next to the nodes are based on 200 bootstrap replicates.
